# Quantifying Intramolecular Binding in Multivalent Interactions: A Structure-Based Synergistic Study on Grb2-Sos1 Complex

**DOI:** 10.1371/journal.pcbi.1002192

**Published:** 2011-10-13

**Authors:** Anurag Sethi, Byron Goldstein, S. Gnanakaran

**Affiliations:** 1 Theoretical Biology and Biophysics, Los Alamos National Laboratory, Los Alamos, New Mexico, United States of America; 2 Center for Nonlinear Studies, Los Alamos National Laboratory, Los Alamos, New Mexico, United States of America; National Institute of Diabetes and Digestive and Kidney Diseases, National Institutes of Health, United States of America

## Abstract

Numerous signaling proteins use multivalent binding to increase the specificity and affinity of their interactions within the cell. Enhancement arises because the effective binding constant for multivalent binding is larger than the binding constants for each individual interaction. We seek to gain both qualitative and quantitative understanding of the multivalent interactions of an adaptor protein, growth factor receptor bound protein-2 (Grb2), containing two SH3 domains interacting with the nucleotide exchange factor son-of-sevenless 1 (Sos1) containing multiple polyproline motifs separated by flexible unstructured regions. Grb2 mediates the recruitment of Sos1 from the cytosol to the plasma membrane where it activates Ras by inducing the exchange of GDP for GTP. First, using a combination of evolutionary information and binding energy calculations, we predict an additional polyproline motif in Sos1 that binds to the SH3 domains of Grb2. This gives rise to a total of five polyproline motifs in Sos1 that are capable of binding to the two SH3 domains of Grb2. Then, using a hybrid method combining molecular dynamics simulations and polymer models, we estimate the enhancement in local concentration of a polyproline motif on Sos1 near an unbound SH3 domain of Grb2 when its other SH3 domain is bound to a different polyproline motif on Sos1. We show that the local concentration of the Sos1 motifs that a Grb2 SH3 domain experiences is approximately 1000 times greater than the cellular concentration of Sos1. Finally, we calculate the intramolecular equilibrium constants for the crosslinking of Grb2 on Sos1 and use thermodynamic modeling to calculate the stoichiometry. With these equilibrium constants, we are able to predict the distribution of complexes that form at physiological concentrations. We believe this is the first systematic analysis that combines sequence, structure, and thermodynamic analyses to determine the stoichiometry of the complexes that are dominant in the cellular environment.

## Author Summary

Many biochemical interactions are mediated by multivalent binding where signaling proteins use relatively weak promiscuous interactions to increase the strength and specificity of complex formation. For a bivalent adaptor protein binding to a multivalent ligand, the tethering of one of the adaptors binding sites to a motif on a multivalent ligand constrains the adaptors second binding site to a region with a high local concentration of ligand binding motifs. Intramolecular equilibrium constants associated with multivalency are difficult to measure. Typically, polymer models are utilized to estimate the enhancement in local concentration and, when the biomolecular equilibrium constants for the individual sites are known, to obtain intramolecular equilibrium constants. However, flexibility of structured regions in proteins that contain the binding motifs restricts the application of simple polymer models for many systems. Here, we develop a hybrid method combining molecular dynamics simulations and polymer models to estimate the intramolecular equilibrium constants. We apply this method to study the multivalent interactions between the widely expressed adaptor protein growth factor receptor bound protein-2 (Grb2) and the nucleotide exchange factor son of sevenless 1 (Sos1).

## Introduction

Grb2 contains one SH2 domain flanked on each side by an SH3 domain [1], [2], each of which forms complexes with multiple polyproline motifs on Sos1. The activation of the Ras signaling pathway requires the recruitment of Sos1 from the cytosol to the plasma membrane where it activates Ras by inducing the exchange of GDP for GTP [3], [4]. This recruitment is mediated by Grb2, which couples Sos1 to phosphorylated receptors and scaffolding proteins that contain sequences of the binding motif for the Grb2 SH2 domain, YXNX. In T cells and mast cells, when the three terminal tyrosines of the scaffolding protein linker for activation of T cells (LAT) are phosphorylated, they become binding sites for the SH2 domain of Grb2. Upon aggregation of T cell receptors on T cells and 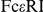 on mast cells, LAT is phosphorylated and aggregates [5]–[7]. When the concentration of Grb2 is sufficiently high compared to Sos1, Grb2-Sos1-Grb2 complexes form and cross-link LAT molecules, unless the concentration of Grb2 is so high that unbound Grb2 fills the binding sites on LAT and blocks cross-linking [6], [8]. Highly specific biomolecular signaling complexes such as the Grb2-Sos1 system often form by combining relatively weak promiscuous interactions. This strategy is widespread with signaling proteins exhibiting a variety of combinations of domains (PH, PTB, SH2, SH3, etc.) that allow them to attach to one or more proteins at multiple sites [9].

Grb2-Sos1 complex formation presents an excellent system for studying the role of multivalency in enhancing the binding affinity. There are four known proline-rich motifs on Sos1 that can bind to the SH3 domains of Grb2 [10], [11]. The effective 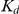 for the formation of a Sos1-Grb2 complex has been measured and is 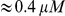 [6], a hundred times smaller than the smallest 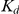 for the binding of a single Grb2 SH3 domain to a proline-rich domain on Sos1. To achieve such an enhancement in its effective equilibrium binding constant, Grb2 must attach to Sos1 through both its SH3 domains. When one SH3 domain is bound to Sos1, the second SH3 domain of Grb2 samples a much higher local concentration of the second binding site than if it were free in solution. The two SH3 domains of Grb2 bind to two of the four proline-rich regions on Sos1 to form a 1∶1 Grb2-Sos1 binary complex. A second Grb2 can bind through both its SH3 domains to this complex to form a Grb2-Sos1-Grb2 ternary complex. At high concentrations of Grb2, the 2∶1 complex is dominant [6]. However, peptide binding studies have shown that only one of the motifs in Sos1 binds strongly to the C-terminus SH3 domain (C-SH3) of Grb2. All the Sos1 motifs bind with moderate strength (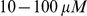) to the N-terminus SH3 (N-SH3) domain [12], [13], raising the question of how the 2∶1 complex forms at physiological conditions.

We present a theoretical study involving the synergistic combination of sequence, structure, molecular dynamics (MD) simulations, and polymer models to determine the stoichiometry of the complexes that dominate the cellular environment. First, a combination of evolutionary analysis of the sequences, and binding energy calculations is used to predict the presence of a new binding motif in Sos1. Secondly, a simple polymer model is used in combination with MD simulations to calculate the enhancement in binding constants due to local concentration. The flexibility of both the modular protein and the disordered region containing binding motifs are taken into account while computing the local concentration effects. We conclude with an evaluation of the stoichiometry of Grb2-Sos1 complexes under physiological conditions and discuss its implications for cell signaling. The approach developed here has applicability beyond the current implementation and provides a framework for handling the multivalency of protein-protein interactions where disordered regions play a significant role.

## Results

### Identification of a new binding motif through evolutionary analysis of Sos

The lack of well-defined structure in the disordered region of the Sos1 protein can, in principle, allow polyproline motifs to bind to SH3 domains of Grb2 in two different orientations [14], [15]. Evolutionary analysis is performed below to identify the presence of any additional polyproline motifs in Sos1 that may bind to Grb2. Previous sequence-based work on Sos1 has concentrated on the four polyproline motifs that bind in the class II (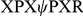) orientation [6], [13]. The C-terminal SH3 domain (C-SH3) of Grb2 binds to class I and class II motifs [12], [16] while the N-terminal SH3 domain (N-SH3) of Grb2 is only known to bind with class II motifs.

**Figure 1:**
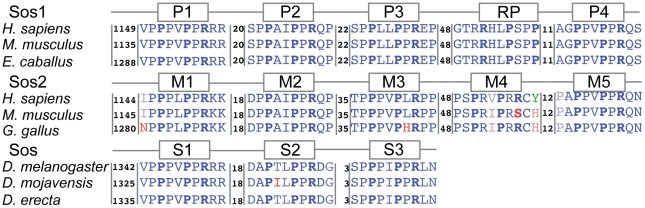
Polyproline motifs in Sos family proteins: These motifs are shown for representative sequences from Sos1, Sos2, and Sos. The sequence is colored to indicate residues that are highly conserved (blue) or variable (red) within each subfamily. The prolines and arginines that are part of the consensus in the class I and class II motifs are shown in bold. The inserted numbers represent the number of amino acids in the linker between the peptides while the first number represents the number of amino acids preceding the first motif in each sequence.

The Sos genes can be divided into three subfamilies - Sos1 and Sos2, found in mammals and higher eukaryotes, and Sos found in flies and mosquitos. Shown in Figure 1, are the four class II motifs on Sos1 (P1 to P4), five class II motifs on Sos2 (M1 to M5), and three class II motifs on Sos (S1 to S3) that Grb2 binds to [17], [18]. These motifs are highly conserved within their respective groups. The first two motifs in Sos1, Sos2, and Sos align in the sequence alignment of the Sos family. In addition, the length of the linker connecting these two motifs is highly conserved in all the Sos proteins (18–20 amino acids). The linker length between the second and third motifs is conserved within their respective groups but is highly variable between the different subfamilies even though P3 and M3 align with each other in the sequence alignment. Finally, the P4 motif in Sos1 aligns well with the M5 motif in Sos2.

An examination of the intrinsically disordered region in the Sos1 sequence reveals a highly conserved class I polyproline motif (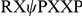) that had not been previously identified. This new motif is marked as RP (residues R1271 - P1277 in *Homo sapiens*) in Figure 1. This region in the Sos1 sequence aligns with the M4 motif present in Sos2 such that the linker length between these two binding motifs is preserved, even though RP is a class I and M4 a class II motif. Based on its conservation within the Sos1 proteins and the linker length conservation across Sos2 and Sos1, we propose that the RP motif on Sos1 is a fifth Grb2 binding motif.

### Versatility of Grb2 adaptor molecule to recognize both class I and II polyproline motifs

To test whether RP can bind to Grb2, we first established and tested a protocol using AutoDock [19] to calculate the binding energies (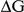) of the experimentally known Sos1 motifs P1 through P4, that bind to the SH3 domains of Grb2. For each peptide, we computationally predict the binding affinities and the sites on the SH3 domains where docking occurs. The binding calculations examine the binding of a full-length SH3 domain with a Sos1 peptide ligand of 9 or 10 amino acids. These ligands have more than 30 torsional degrees of freedom, while AutoDock is most reliable when the ligand has less than 10 degrees of freedom [20]. However, because the binding of the Sos1 peptides to the SH3 domains of Grb2 is enthalpically driven [6], we have neglected the conformational flexibility in the backbones of the Sos1 peptides, which substantially reduces the ligand's degrees of freedom.

**Figure 2:**
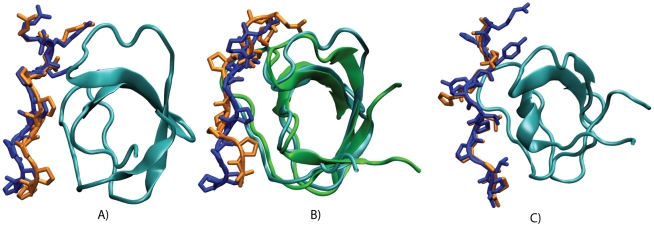
Validation of binding of RP motif to C-SH3. Comparison of the binding sites predicted (in orange) for P1 and the experimental binding site (in blue) for P1 to the (A) NMR structure of the N-SH3 domain (in cyan) (PDB ID 1AZE), and (B) a frame from the molecular dynamics simulation of C-SH3 domain (green). (C) The predicted binding site (orange) for RP peptide to C-SH3 is compared to the NMR structure of the domain bound with a class I peptide (in blue) (PDB ID 1IO6).

Blind predictions of the binding sites and 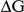 of motifs P1 to P4 in Sos1 with the N-SH3 domain of Grb2 display reasonable agreement with experimentally determined binding sites and energies [13] (Table 1 and Figure 2A). The calculations predict, as has been observed [13], that all four Sos1 peptides are capable of binding to N-SH3 in the class II orientation at the polyproline motif binding site. In Figure 2A, the theoretical prediction for the binding site of P1 on N-SH3 is compared with the experimentally determined binding site. The predicted conformation with the lowest binding energy displayed a RMSD of 2.04 Å for all non-hydrogen atoms with respect to the NMR structure (PDB ID 1AZE [21]).

**Table 1:** Binding Energies of motifs to N-SH3 domain of Grb2.

Motif	Sequence	Comp.	Comp.	Expt.	Expt.
		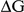	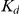 ( 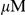 )	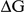	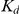 ( 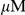 )
P1	PPPVPPRRR	−7.1	6.2	−6.02	39
P2	PPAIPPRQP	−7.1	6.2	−5.80	56
P3	PPLLPPREP	−5.1	182	−5.36	117
P4	GPPVPPRQS	−6.0	40	−5.58	82
RP	TRRHLPSPP	−5.2	153	–	–

Comparison of computational (Comp.) and experimental (Expt.) binding energies (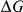) (in kcal/mol) and dissociation constants (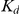) of different motifs on Sos1 to N-SH3 domain of Grb2.

Unlike for the Grb2 N-SH3 domain, the only high-resolution structure available for a peptide bound to the Grb2 C-SH3 domain is for a class I motif. Conformational changes are expected when a SH3 domain binds to a class I versus a class II motif [15]. As P1 to P4 are class II motifs, the protocol for blind binding predictions of the C-SH3 domain binding to P1 through P4 motifs required an additional step for generating the backbone conformations for the ligands and the conformation of the C-SH3 domain. A molecular dynamics (MD) simulation of the C-SH3 domain bound to a strong binding peptide P1 was used to generate conformations for the backbone of the peptides and the C-SH3 domain. These conformations were then used during the blind binding predictions of the class II motifs in Sos1 to C-SH3. MD simulations have previously been used to produce good candidate conformations for binding energy predictions as, for example, in predicting novel inhibitors for RNA-editing ligases [22].

As seen in Figure 2B, the predicted binding sites for P1 on C-SH3 and N-SH3 are similar. The larger variation in the binding site conformation in Figure 2B compared to that in Figure 2A arises, in part, because the conformations for the backbone of the peptide and the C-SH3 domain used in the binding energy calculations vary from those of the experimental structure for the N-SH3 domain bound to P1. Note that compared to the peptide motifs used in the docking calculation for binding of the peptides to the Grb2 N-SH3 domain, an additional amino acid at the N-terminus of these peptides was needed for accurate binding predictions to the C-SH3 domain. This extra amino acid was particularly critical for predicting the correct C-SH3 domain binding site for P3. The predicted 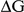 for P1 through P4 motifs on C-SH3 agree reasonably well with the measured quantities as shown in Table 2. Also consistent with experiment, the 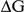 predicted for the binding of the P1 motif to C-SH3 is greater than the 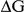 for the domain binding to P2, P3, and P4 motifs.

**Table 2:** Binding Energies of motifs to C-SH3 domain of Grb2.

Motif	Sequence	Comp.	Comp.	Expt.	Expt.
		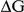	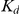 ( 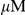 )	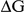	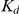 ( 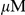 )
P1	VPPPVPPRRR	−7.5	3.2	−5.29	125
P2	SPPAIPPRQP	−4.9	255	−3.87	1396
P3	SPPLLPPREP	−4.5	501	−3.78	1718
P4	AGPPVPPRQS	−4.3	702	−3.92	1318
RP	GTRRHLPSPP	−5.1	182	–	–

Comparison of computational (Comp.) and experimental (Expt.) binding energies (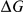) (in kcal/mol) and dissociation constants (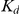) of different motifs on Sos1 to C-SH3 domain of Grb2.

It is worth mentioning that we did consider the binding of P1–P4 peptides with flexible backbones to the SH3 domains. However, these calculations led to convergence issues with AutoDock due to the relatively large number of degrees of freedom of these flexible peptide fragments. The program was not able to discriminate between the experimentally known binding site and another binding site on the opposite side of the SH3 domain. Still, the free energy for binding to the experimentally determined site was comparable to the binding free energy obtained in Tables 1 and 2 (with a difference of approx. 0.5 kcal/mol). To take the backbone flexibility into account we used an alternate approach. We performed the AutoDock calculations with ten different conformations from MD simulations for each of the peptides binding to the N-SH3 and C-SH3 domains of Grb2 (Table S1). Each conformation of the peptide bound SH3 domain exhibited some variability in the backbone conformation both in the peptide and the SH3 domain. Even though the means of the calculated binding energies were similar to what we originally reported, the variance of the energies did capture the influence of backbone flexibility. The variation in the calculated binding energies is larger for P1 binding to the C-SH3 domain than for any peptide-SH3 domain binding combination we tested. We expect this binding interface to be more fluxional due to the electrostatic nature of the three terminal arginines and its interactions with glutamic acids in the C-SH3 domain.

Furthermore, to ensure that this approach is sensitive to the binding specificity of the SH3 domains, we mutated the three arginines at the C-terminus of the P1 motif to alanines. This mutated peptide is expected to present a low binding affinity for the motif because of the absence of the terminal arginine in the class II 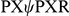 motif (i.e., a true negative versus a false positive test) [16]. The theoretically predicted binding energy for the mutated motif to both N-SH3 and C-SH3 domains (−4.8 and −4.7 kcal/mol respectively) was found to be lower than the binding energies of the four wildtype motifs on Sos1 (Tables 1 and 2). Interestingly, for the P1 mutated sequence, there was a change in the predicted position of the binding site. The mutated form is predicted to bind on the opposite face of the SH3 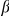-barrel than the motifs P1 to P4. Thus, this protocol is sensitive to the specificity of the SH3 domains and can be used to validate whether the RP motif will bind to the SH3 domains.

The same protocol for estimating 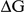 was then used to test whether RP can bind to the N-SH3 and C-SH3 domains of Grb2. This protocol predicts that the newly identified class I motif RP is capable of binding to the N-SH3 and C-SH3 domains of Grb2 with similar affinities as P3. As shown in Figure 2C, the binding site and orientation predicted for RP are similar to the experimentally determined conformation of a class I motif bound to C-SH3 (PDB ID 1IO6 [16]). All-atom MD simulations of N-SH3 bound to the RP motif were carried out to evaluate whether the N-SH3 forms a stable complex with RP. Consistent with the binding energy calculations, the peptide remains bound to N-SH3 after 300 ns of MD simulation, and all the critical interactions between the peptide and SH3 remain intact through this period.

The main purpose of the extensive binding energy calculations provided above is to show that the newly identified RP motif in Sos1 binds to the SH3 domains of the Grb2 with similar affinities as some of the other poly-proline motifs from Sos1. AutoDock, which was used to compute affinities, is less reliable at predicting the values of equilibrium constants than at predicting binding sites [23]. As can be seen from Tables 1 and 2, binding calculations predict consistently higher affinities compared to the experimentally determined values. However, the trends between experimentally and computationally determined binding affinities are similar. Based on these trends, we expect the affinity of RP to be of the same order of magnitude as that of P3. In the ensuing calculations of the intramolecular equilibrium constants, we will use the measured affinities for single site equilibrium constants and take the affinities of RP to be the same as P3.

**Figure 3:**
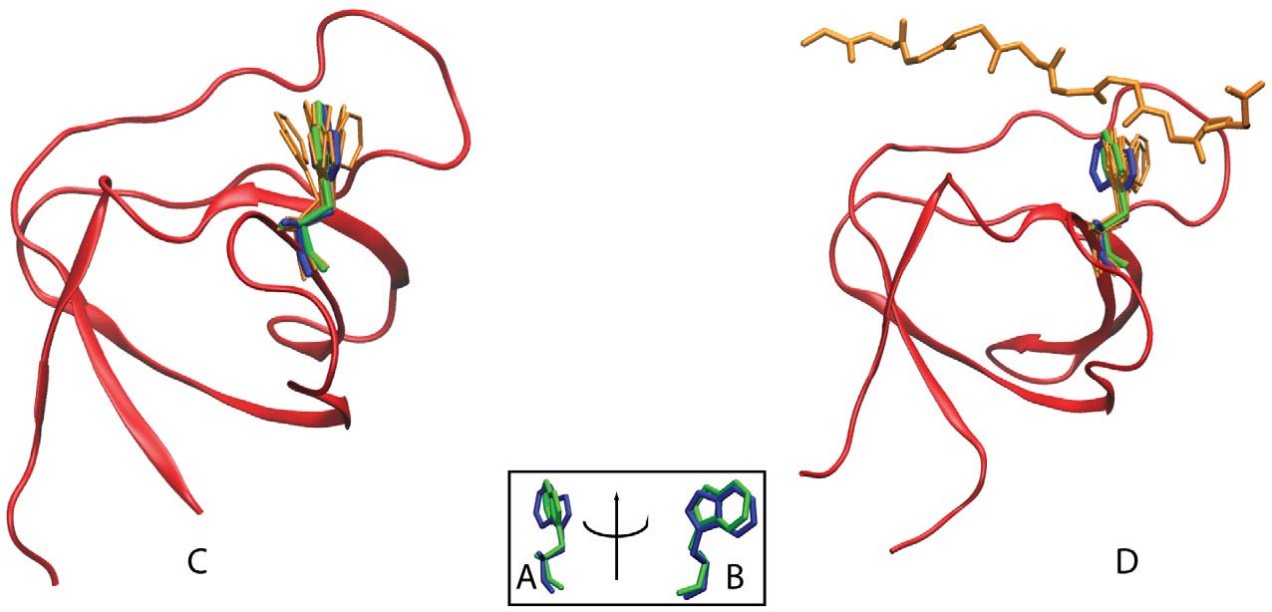
Flexibility of the conserved tryptophan switch in Grb2. (A) and (B) show the expected conformations of the W switch for class I (blue) and class II (green) peptide bound SH3 domains based on static x-ray structures (PDB IDs 1CKA and 1ABO respectively). In MD simulations of the C-SH3 of Grb2, the flexibility of the W switch is greater than the flexibility required to bind both class I and II peptides in the (C) *apo* and (D) peptide bound Grb2 simulations.

Given that the class I ligand RP can bind to the SH3 domains of Grb2, we examined the N-SH3 and C-SH3 domains for the presence of any structural signatures that might indicate why they are able to bind to both class I and class II ligands. According to previous studies [15], the orientation of a conserved tryptophan switch (W37 and W193 in Grb2) in the SH3 binding pocket determines specificity based on whether a SH3 domain is capable of forming a specific hydrogen bond with the backbone of class I or II motifs. On locally aligning all class I and class II-binding SH3 domains [15], we find significant differences in orientation of the W switch between the two classes (Figure 3A and B). An SH3 domain that binds to both class I and class II motifs has the inherent flexibility to exist in both class I and class II binding orientations in the absence of a ligand [15].

In order to estimate whether the W switches in Grb2 has the inherent flexibility to bind to both class I and class II ligands, all-atom MD simulations of Grb2 were carried out in explicit water in the presence (Figure 3) and absence (Figure 3) of a bound peptide. We compared the conformation of the conserved switches (W37 and W193 in N-SH3 and C-SH3 respectively) in Grb2 during the simulations with the conformation of the W switch in a class II (PDB ID 1ABO [24]) and a class I (PDB ID 1CKA [25]) peptide binding orientation. Here, each frame from the trajectory was overlapped with the class I and II binding SH3 domains based on a local alignment involving the backbone atoms of residues *n*−2 to *n*+2 where *n* refers to the W residue. The W switch is highly flexible and is capable of forming hydrogen bond interactions with class I and class II polyproline motifs as shown in Figure 3C. Despite the highly fluxional character in the conformations of W193 in the C-SH3 domain bound to P2, the hydrogen bond between the side chain of W193 and the backbone of the peptide is maintained in most of the frames of the simulation. Hence, we find that orientations of the W switch of N- SH3 and C-SH3 are fluxional enough to bind both class I and II polyproline motifs in Sos1.

### Multivalent binding of both Grb2 SH3 domains to Sos1

We have separately characterized the binding of each motif in Sos1 to the SH3 domains of Grb2. As Grb2-Sos1 forms a multivalent complex, these interactions are influenced by local concentration effects after one motif in Sos1 binds to Grb2. We wish to calculate the effective local concentration (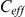) of Sos1 motifs that a free SH3 domain on Grb2 experiences when its other SH3 domain is bound to a motif on Sos1. The concentration of Sos1 is assumed to be sufficiently low so that cross-linking of two Sos1 by a single Grb2 can be neglected.

**Figure 4:**
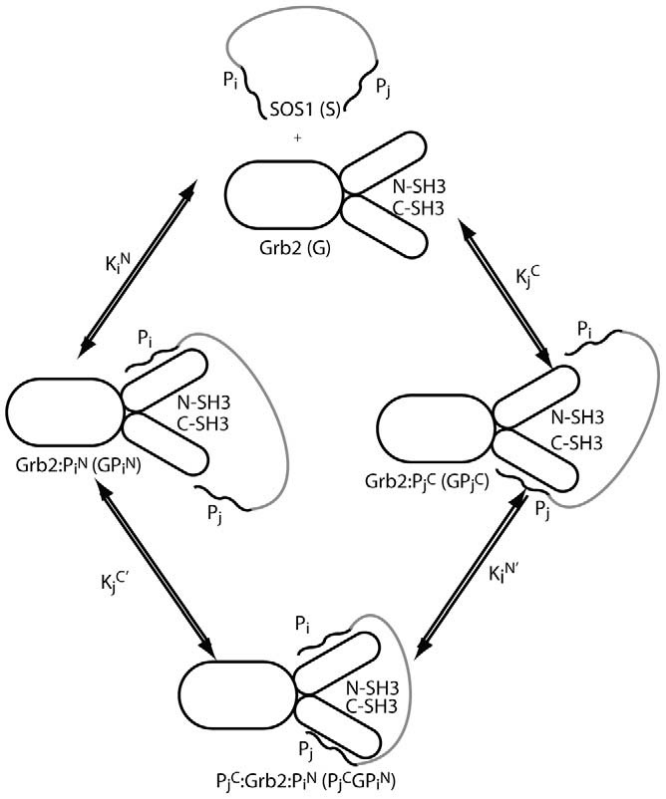
The possible steps in the binding of two polyproline binding sites, 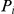 and 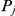, on Sos1 to the N-SH3 and C-SH3 domains on Grb2 to form a doubly bound 1∶1 complex of Grb2 and Sos1. 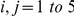
, represents the peptides P1 through P4 and RP respectively and 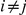.

The binding of two motifs on Sos1 to the two SH3 domains of Grb2 follows the scheme shown in Figure 4. There are two steps in the multivalent binding of Grb2 to Sos1 - the first is intermolecular while the second is intramolecular. We define 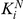 and 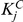 as the equilibrium binding constants for the binding of motifs 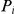 and 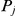 to the N-SH3 and C-SH3 domains of Grb2 respectively.
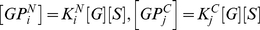
(1)where 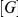, 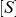, and 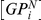 are the concentrations of unbound Grb2, unbound Sos1, and Grb2 bound to the 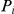 motif of Sos1 with its N-SH3 domain. 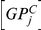 is similarly defined. In the case where 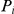 and 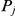 are motifs in the same Sos1 molecule tethered by a disordered protein segment,
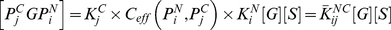
(2)where 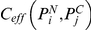 is the effective concentration of motif 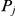 that the C-SH3 experiences when the N-SH3 of Grb2 is tethered to 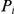 on Sos1 and 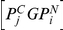 is the concentration of doubly bound Grb2. 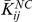 is defined as the effective equilibrium constant for the simultaneous binding of motifs 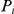 and 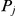 on a single Sos1 molecule to Grb2 and is given by:
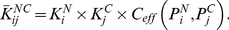
(3)


Note that the effective binding constant of motifs 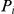 and 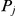 in Sos1 to the corresponding domains in Grb2 is independent of the order of binding of both motifs as required by detailed balance. While the intermolecular binding constant 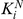 and 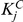 are known experimentally [13], the intramolecular equilibrium constants, 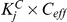 and 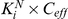, have not been measured and it is difficult to measure these parameters directly.

### Hybrid MD-polymer theory model for estimating effective binding constants of Grb2-Sos1 complex

**Figure 5:**
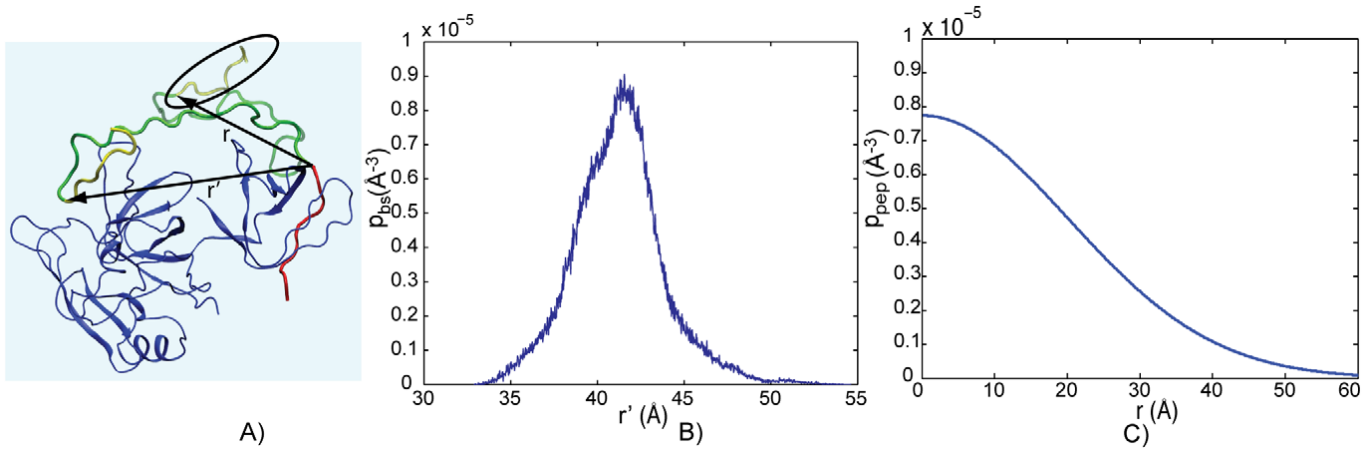
Example calculation for 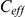. In (A) the two motifs that bind to Grb2 (blue) are shown in red and yellow while the linker in Sos1 connecting them is shown in green. After one of the motifs binds to Grb2, the local concentration (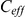) of the other Sos1 motifs that the free SH3 domain of Grb2 feels increases (circled) as Sos1 is tethered to Grb2. In order to calculate 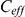, we used MD simulations (B) to determine the distance between the 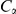 atoms at the two ends of the motif when they are bound to Grb2 (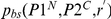) and the WLC model (C) to determine the probability densities of the distance between the 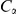 atoms at the two ends of a linker (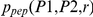).

For a Grb2 with its N-SH3 domain bound, 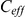 is proportional to the probability of finding the C-SH3 of Grb2 and the 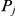 motif on Sos1 together in the same region of space. As shown in Figure 5 for binding of P1 and P2 to N- and C-SH3 domains of Grb2 respectively, we define 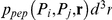 to be the probability of finding 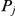 on the tethered Sos1 at the position 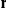 in the volume 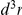 and 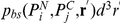 to be the probability of finding the C-SH3 domain on the tethered Grb2 at the position 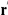 in the volume 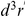. Assuming that the linker region does not interact with the SH3 domains in Grb2, 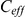 is given by the expression [26], [27]:
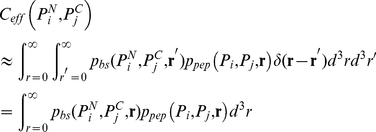
(4)


A hybrid approach combining a polymer model and MD simulations is used to obtain expressions for the probability densities in Equation 4. Ignoring any interactions between the linker and Grb2, 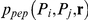 is obtained by treating the span of Sos1 from 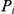 to 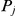 as a polymer described by the worm-like chain (WLC) model [28], [29]. When the length of the polymer is much longer than its persistence length (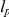), this model predicts that:
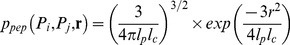
(5)where 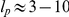 Å for unfolded peptides [30]–[32] and 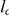 is the contour length of the peptide (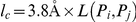 where 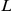 is the number of amino acids in the linker connecting motifs 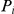 and 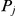). The probability density 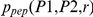 is shown in Figure 5C. Experimental studies indicate that the persistence length for native unstructured proteins is a weakly increasing function of the length of the protein [32] and 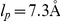 for a 203 amino acid disordered region.

**Figure 6:**
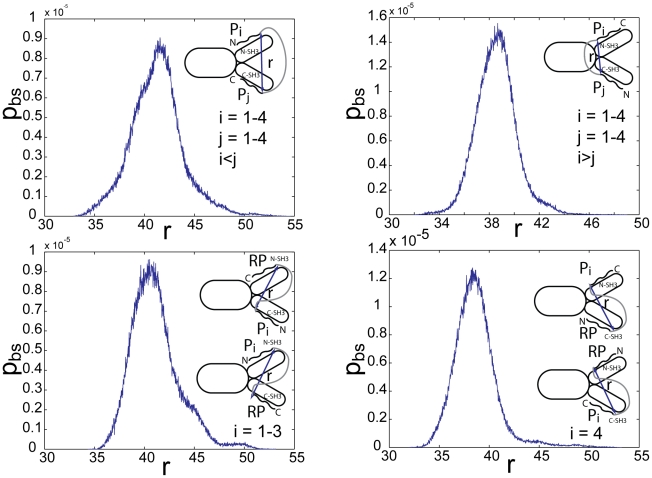
The probability density for distance between binding sites (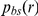) in Sos1 bound to Grb2. Depending on the 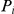 and 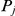 motifs bound to Grb2, one of four different probability distributions are used (explained in inset of figures).

To obtain the probability density for the vector distance between the SH3 domain binding sites in Grb2, Zhou [26] used a composite WLC model representing two flexible linkers separated by a rigid rod to model the effect of the SH2 domain in Grb2, but recognized that detailed effects such as excluded-volume and steric interactions were ignored in this approach and that MD or Monte Carlo simulations to obtain 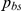 might be warranted. To estimate 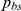, we used a 400 ns MD simulation of Grb2 bound to P1 and P2 in the absence of a linker (see Figure 5B for an example). This probability density will depend on what type of polyproline ligand (class I or II) each motif is, and on the order of the motifs 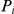 and 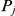 in the sequence of Sos1 (see Figure 6).

An intrinsic problem with obtaining the probability distribution using MD simulations is that it may not reflect the true distribution because of limited conformational sampling. To test for convergence, we split the 400 ns MD simulation into two halves of 200 ns each. We calculated 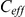 and 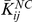 based on both halves of the MD simulation separately. As the values of 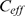 and 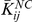 are nearly the same between both halves of the MD simulation. Even though the MD simulations show that the probabilistic density of the distance between the binding sites (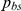) tends to converge on the time scale of 200 ns, any global conformational changes on time scales longer than sub-microseconds will influence this distribution. The effect of these global conformational changes in 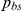 can be incorporated by using coarse grained MD simulations such as the method proposed in [33].

In Table 3, we list the calculated effective concentrations for all motifs on Sos1 that a SH3 domain on Grb2 experiences when its second SH3 domain is bound on the same Sos1. Almost all the 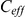 are in the mM range as was also obtained in [26] for the binding of Grb2 to a small bivalent ligand. In comparison, the cytoplasmic concentration of Sos1 in Jurkat cells is 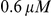 [8]. In Table S2, we show that the 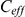 is estimated to be in the mM range when the probability density of the distance between the binding sites (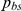) is approximated using a set of delta functions.

**Table 3:** Intramolecular binding of two motifs in one Sos1 molecule to the two SH3 domains of Grb2.

		Bound to N-SH3 domain				
		P1	P2	P3	RP	P4
**Bound to C-SH3 domain**	**P1**	-	2.1 (3.4)	1.6 (9.2)	0.8 (19)	0.6 (16)
	**P2**	1.7 (33)	-	2.1 (77)	1.0 (156)	0.9 (133)
	**P3**	1.4 (47)	1.7 (56)	-	1.6 (129)	1.3 (112)
	**RP**	0.8 (88)	1.0 (92)	1.6 (129)	-	1.4 (111)
	**P4**	0.6 (84)	0.8 (91)	1.2 (132)	1.4 (117)	-

Effective concentration of the motif (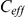 in mM) near the second binding site when one of the motifs is bound to the appropriate SH3 domain. The effective dissociation constant (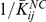 in 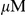) of both motifs in Sos1 binding to Grb2 is written in parentheses.

From the 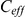 in Table 3, and the experimentally measured equilibrium constants for the binding of peptides P1 to P4 on Sos1 to the N-SH3 and C-SH3 of Grb2 in Tables 1 and 2 [13], one can quantify the enhancements in binding affinities that result from Grb2 having two SH3 domains that bind to multiple sites on the same Sos1. Listed in Table 3 are the effective dissociation constants calculated from Eq (3) for the formation of doubly bound Grb2. The single site affinities for the binding of SH3 domains to the RP motif have not been measured. To calculate an effective dissociation constant, we take the binding affinities of RP to the SH3 domains to be the same as those between P3 and the SH3 domains. Note that P3 is the poorest binder to Grb2 of the four motifs [13].

McDonald et al. showed that C-SH3 binds strongly to P1, with a dissociation constant 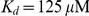, but binds poorly, if at all, to P2, P3 and P4 (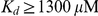) [13]. As a result, in Table 3 the strongest binding is predicted to occur for doubly bound Grb2 with its C-SH3 domain bound to P1, with these effective binding constants being greater than the binding constants for singly bound Grb2 to any of the peptides. Thus, when the Grb2 concentration is much lower than the Sos1 concentration, we expect Grb2 to be doubly bound to Sos1 with its C-SH3 domain bound to the P1 domain.

### Comparison with binding measurements on Grb2-Sos1 complex

**Figure 7:**
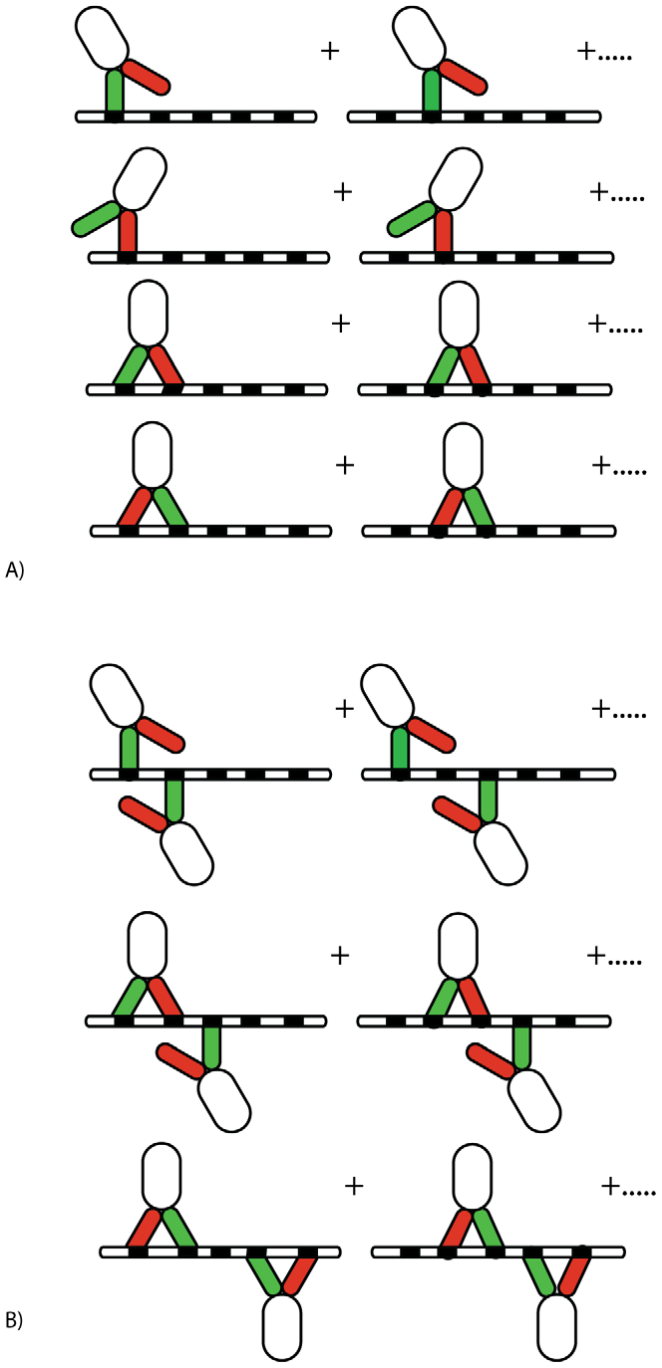
Combinations of 1∶1 and 2∶1 Grb2-Sos1 complexes. A) In the 1∶1 Grb2-Sos1 complex, Grb2 can be bound to Sos1 through one or both SH3 domains (shown in green and red) to polyproline motifs (black boxes) on the unstructured C-terminus tail of Sos1 (represented by an oval). B) In the 2∶1 Grb2-Sos1 complex, each Grb2 can be bound to Sos1 through one or both SH3 domains to the §polyproline motifs on Sos1. The SH2 domain of Grb2 is shown as a white oval shape connected to the SH3 domains.

The binding constants for Grb2-Sos1 complex formation have been measured [6]. The 1∶1 Grb2-Sos1 complex is expected to consist of multiple species due to the presence of multivalent interactions between Grb2 and Sos1. A Grb2 in a 1∶1 Grb2-Sos1 complex is bound either through one or both of its SH3 domains (Figure 7A). From Eqs. (1) and (3):
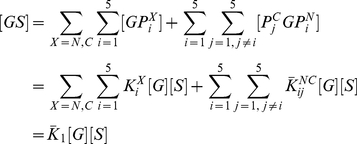
(6)where
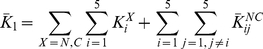
(7)

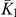
 is the effective equilibrium constant for the formation of a Grb2-Sos1 complex. Because five binding sites on Sos1 can interact with the two Grb2 SH3 domains, there are 30 different possible 1∶1 Grb2-Sos1 complexes.

A 2∶1 Grb2-Sos1-Grb2 complex can be composed of a Sos1 molecule bound to two singly bound Grb2, to a singly and a doubly bound Grb2, or to two doubly bound Grb2 (Figure 7B). The overall concentration of the 2∶1 Grb2-Sos1-Grb2 ([GSG]) complex is:
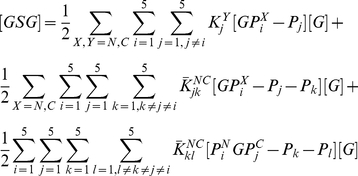
(8)


The factor of two appears in the denominator because the Grb2 molecules are indistinguishable. In other words, the order of the different Grb2 molecules binding to the peptides does not matter as long as the same complex is formed. This equation can be rewritten as:
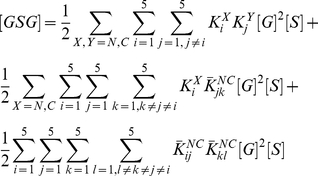
(9)or in other words:
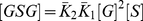
(10)where 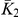 is the effective equilibrium constant for the binding of a Grb2 from solution to a Grb2-Sos1 complex to form a Grb2-Sos1-Grb2 complex:
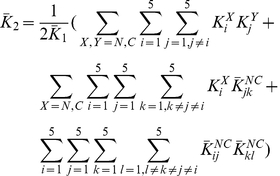
(11)


To make predictions about the binding of Grb2 to the complete polyproline rich domain of Sos1 (1117–1319) we must estimate the values of the unknown equilibrium constants for the binding of the N- and C-SH3 domains of Grb2 to RP on Sos1. As in calculating the effective concentrations in Table 3, we took these equilibrium constants to be the same as for binding to the P3 peptide. We predict that 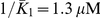 and 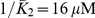. Chook et al. [34] found that the full Sos1 molecule, immobilized on a Biacore chip, bound Grb2 with a stoichiometry of 1∶1 and a dissociation constant of 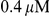, about a factor of three lower than our calculated value of 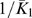.

The prediction of the computed effective equilibrium constant within a factor of four of the measured value is encouraging considering the approximations and the complexity of the system. In addition to the approximations associated with 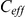 (Eq. 4) as discussed above, the difference in single site affinity between a motif embedded in Sos1 and one that binds in isolation may have contributed to the observed discrepancy. The single site affinities used in our calculation are based on measurements of 12 amino acid length peptides (lacking flanking sequences) to the SH3 domains of Grb2. However, one can expect changes in affinities due to flanking sequences [35], [36]. The flanking regions may affect the binding affinity of each motif by a different factor. In such a scenario, the bivalent binding constants, which involve two motifs, will be modified by the product of the corresponding two factors (Eq. 3). However, we make the simplifying assumption that the flanking regions do not modify the binding affinity of the motifs to the SH3 domains in the full length Grb2 and Sos1. Furthermore, we have neglected any allosteric communication between the two binding sites in Grb2 that could either increase or decrease the affinity for bivalent binding to Sos1 but have no effect on the monovalent binding affinities.

Importantly, these effective equilibrium constants can be used to calculate, for example, the fraction of 1∶1 complexes that are composed of a singly or doubly bound Grb2. The fraction with Grb2 singly bound is just the ratio of the first term in Equation 7 divided by 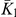. We predict that 10% of the Grb2-Sos1 complexes have Grb2 bound through a single SH3 domain while the remaining 90% have Grb2 bound through both its SH3 domains. Similarly, we predict that 68% of the Grb2-Sos1-Grb2 complexes have both Grb2 doubly bound to Sos1, 27% have one Grb2 doubly bound and one singly bound, and 5% have both Grb2 singly bound.

### Comparison with binding measurements on Grb2-Sos1NT complex

As the equilibrium binding constants for the binding of the N- and C-SH3 domains of Grb2 to RP have not been measured, it is difficult to judge the accuracy of the model from predictions that require knowledge of these equilibrium binding constants. Houtman et al. [6], [37] have determined the equilibrium constant for the binding of Grb2 to a 96 amino acid N-terminal fragment of Sos1 (Sos1NT) that contained only the polyproline-rich motifs, P1, P2 and P3. Using isothermal titration calorimetry (ITC) they found the stoichiometry of the binding of Grb2 to Sos1NT to be 1∶1 with a 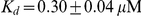. Our model calculations predict a 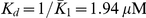 for a 96 amino acid unstructured protein with 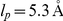 [32]. However, since Sos1NT has three Grb2 binding sites the possibility arises that at sufficiently high ratios of Grb2 to Sos1NT, binding stoichiometries of 2∶1 (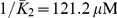) and possibly 3∶1 may occur. In order to fit all the products to a 1∶1 complex, we predict the 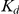 to be:
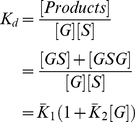
(12)In Figure 5 of reference [37], the interaction of Grb2 with Sos1NT was studied by titrating Grb2 to a maximum concentration of 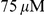 against Sos1NT, reaching a molar ratio of Grb2∶Sos1NT of 2–2.25. For these experiments, where the free concentration of Grb2 is always less than 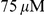, we predict that the effective stoichiometry of the Grb2-Sos1 complexes is 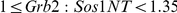. When the contribution of 2∶1 binding is taken into account, we calculate the effective 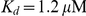, a factor of four higher than the measured value [6], [37].

Using ITC Houtman et al. [6] also determined the equilibrium constant for binding of Grb2 to a C-terminal fragment of Sos1 (Sos1CT) that contained P4 and RP. They found the stoichiometry to be 1∶1 with 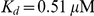. Our model calculations predict a much higher value, a 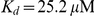. For this calculation we took the values of the unknown binding affinities of RP for the N- and C-terminal SH3 domains of Grb2 to be the same as the measured values of P3, the proline-rich motif that is the weakest binder of Grb2. The discrepancy between the measured and calculated 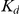 values for Grb2 binding to Sos1CT suggests that we have underestimated the RP affinities, although other approximations that we have indicated are likely to also contribute to the discrepancy.

### Stoichiometry of Grb2 and Sos1 complexes at cellular concentrations

**Figure 8:**
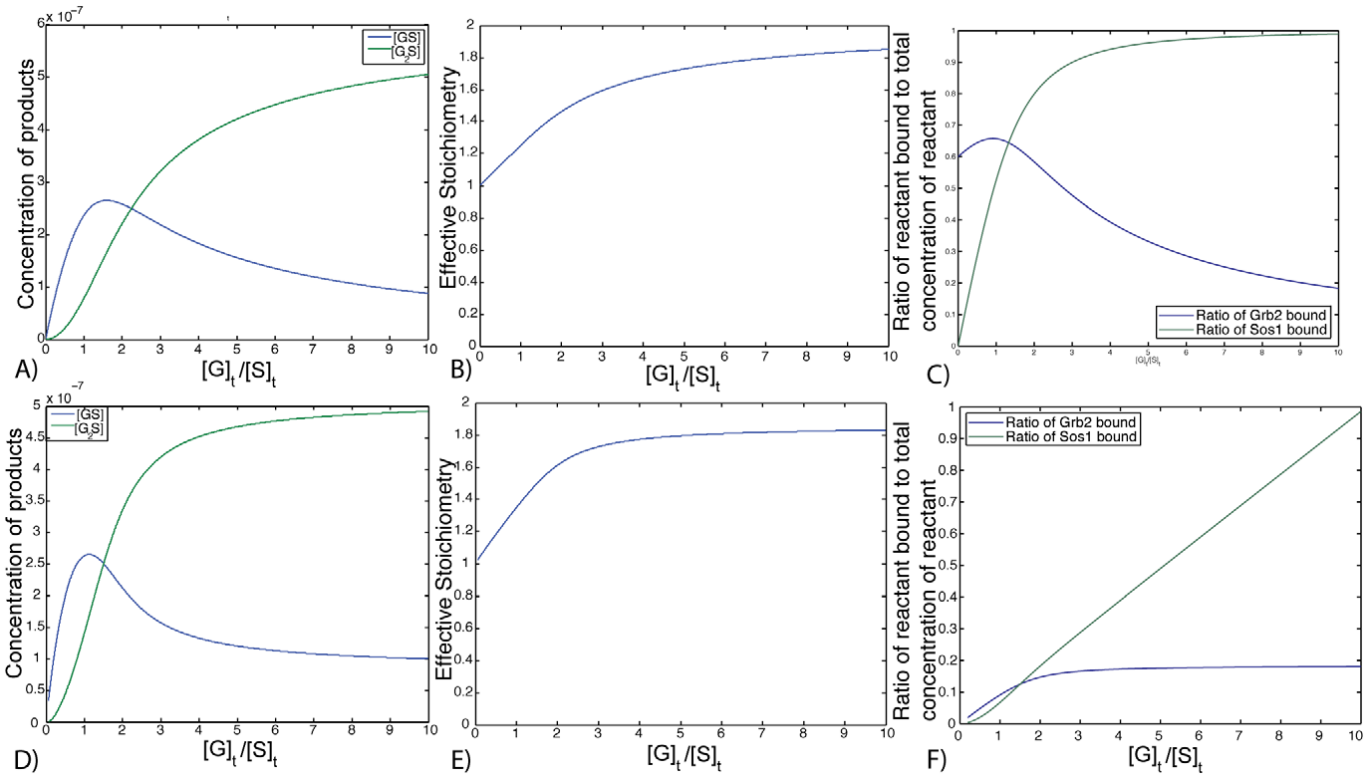
Stoichiometry of Grb2-Sos1 complexes at cellular concentrations. The concentration of Grb2 is increased at constant concentration of 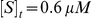 in A, B, and C. The concentration of Sos1 is varied at constant concentration of 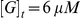 in D, E, and F. The concentration of products (A, D), effective stoichiometry (B, E), and ratio of bound to total concentrations of Grb2 and Sos1 (C, F) are plotted against the ratio of the concentration of reactants (Grb2 and Sos1).

When T cells are activated the transmembrane scaffolding protein LAT is rapidly phosphorylated [38], followed by the formation of large aggregates of LAT [5], [6]. The aggregation is mediated by Grb2 [6]. Fully phosphorylated LAT has three binding sites for the SH2 domain of Grb2 [39]. LAT aggregation is a result of Grb2-Sos1-Grb2 complexes bridging two LAT molecules; each Grb2 in the complex bound to a separate LAT molecule through its SH2 domain. If aggregates containing large numbers of LAT are to form, the cytosolic concentrations of Sos1 and Grb2 must favor formation of 2∶1 complex. In Jurkat E6.1 cells, the concentration of Grb2 is 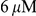, which is 10 times higher than the concentration of Sos1 in these cells [8]. Assuming only 1∶1 and 2∶1 Grb2-Sos1 complexes form, the fraction of complexes containing two Grb2, 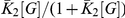, equals 0.83 for the measured value for 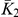 (see Figure 8). This is based on the experimental dissociation constant, 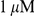, for the formation of the Grb2-Sos1-Grb2 complex from the Grb-Sos1 complex. Existence of such a large number of the complexes containing two Grb2 molecules are predicted to lead to the formation of large aggregates of LAT [7]. For our calculated value of 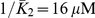, we predict that 0.27 of the complexes would contain two Grb2. This seems low, suggesting that our value for 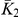 is an underestimated, or that the measured concentration of Grb2 in Jurkat T cells is too low.

## Discussion

Many signaling proteins use multivalency, combining relatively weak promiscuous interactions to increase the strength and specificity of complex formation [40],[41]. Intramolecular equilibrium constants associated with multivalency are difficult to measure and mostly remain undetermined. Typically, polymer models are utilized to fill the gap, when the biomolecular equilibrium constant for the individual sites are known [26], [27], [42], [43]. At the heart of the method is the calculation of the effective concentration of a binding motif on one protein, that the binding site on the second protein experiences, when the two proteins are tethered. A simple polymer model, the WLC, has been used to characterize the flexibility of the portions of the proteins that participate in forming the intramolecular bond [26], [27], [42], [43]. Barua et al. [44] analyzed a variety of *in vitro* studies of the binding of the tandem SH2 domains on the phosphoinositide 3-kinase (PI-3) p85 regulatory domain to its bisphosphorylated binding site in the cytoplasmic domain of the platelet-derived growth factor 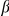
–receptor (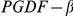). They concluded that the effective concentration for formation of the intramolecular bond was three orders of magnitude lower than predicted by the WLC model and that factors other than peptide dynamics, such as the conformational dynamics of the tandem SH2 domains, impose structural constraints on the interaction. Thus, using the WLC model to predict the spatial distribution of binding sites restricts the application of polymer based methods to unstructured proteins or regions of proteins that are disordered.

We have chosen a hybrid MD-polymer approach to study the complex formation of a highly structured adaptor protein containing two SH3 domains, Grb2, with a disordered region of the protein Sos1 that contains at least four, and possibly five, binding sites for the SH3 domains of Grb2. Our hybrid MD-polymer methodology calculates 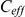 by taking into account the flexibilities of the structured domains of Grb2 with MD simulations and the unstructured Sos1 with a simple polymer model. We expect that the WLC model provides a reasonable description of the spatial statistics of the linker connecting any two motifs in the disordered segment of Sos1. The MD simulation of Grb2 in explicit water provides an accurate description of the probability density for the distance between the two SH3 binding sites when one site is bound. We show that the local concentration of the Sos1 motifs that a Grb2 SH3 domain experiences is approximately 1000 times greater than the cellular concentration of Sos1. Unlike in the studies of Barua et al. [44], binding studies on Grb2 and Sos1 suggests that the three orders of magnitude enhancement in local concentrations predicted using the hybrid method might be an underestimate.

As all polyproline motifs occur in the disordered region of Sos1, the inherent flexibility gives rise to a large number of molecular species in Grb2-Sos1 complexes. We used the measured single site equilibrium constants for the binding of the separate Grb2 SH3 domains to the 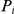 peptides [13] to estimate the intramolecular equilibrium constants of these species contributing to complex formation. The calculated 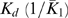 for the entire Sos1 molecule is a factor of three higher than the measured value [34], while for the Sos1 fragment containing the first three binding motifs, 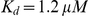, a factor of four higher than the measured value [6], [37]. Lack of sampling and inaccuracies in the force field in the MD simulations, the simplicity of the WLC model, neglect of the interactions between linker and Grb2, and neglect of any allostery between N-SH3 and C-SH3 domains in Grb2, all may introduce errors in 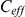 and contribute to the weaker binding predicted than observed. Also, the single site affinity values we use in our calculations, which come from binding studies using 12 amino acid length peptides lacking flanking sequences [13], may differ from the values that would be obtained for binding motifs embedded in Sos1 [35], [36]. Nevertheless, we were able to use a purely computational approach, in the absence of any additional parameters, to calculate an effective equilibrium constant for binding of Grb2 to Sos1 to within an order of magnitude of the experimental value. We are optimistic that such an approach could be used to estimate the effective equilibrium constants for multivalent complexes in the absence of experimental information.

**Figure 9:**
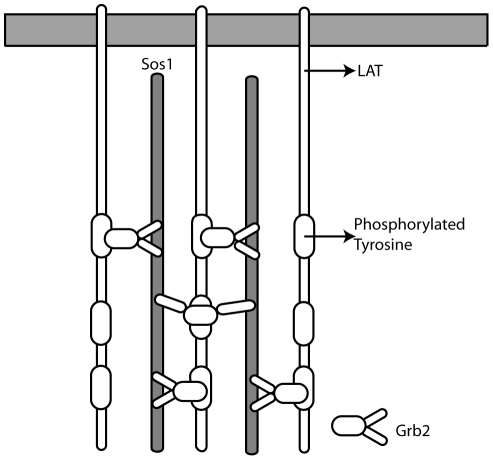
Crosslinking of Sos1 molecules by Grb2 at the membrane.

Finally, we want to comment on the nature of complexes that form under physiological concentrations and on the impact of the newly predicted fifth motif in Sos1 on downstream signaling. Binding studies of Grb2 to Sos1 under physiological conditions suggests that the valence of Sos1 for Grb2 is two and that a bound Grb2 has both its SH3 domains attached to Sos1 [6]. Our calculations clarify why, over the concentration ranges studied, this is a reasonable description of the binding. For these concentrations, only 1∶1 and 2∶1 complexes of Grb2 are predicted to form with measurable concentrations. The newly identified fifth proline-rich motif on Sos1 could lead to additional cross-linking. As the equilibrium constants for the Sos1 motifs to SH3 in Grb2 are low, 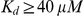 [13], and the concentration of Sos1 in Jurkat T cells is 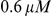 [8], we expect cross-linking of two Sos1 by a single Grb2 to be negligible in the cytosol. However, the fifth site might play a role after Sos1 is brought close to the membrane. Once T cells are stimulated and Sos1 is recruited to LAT, the effective Sos1 concentration just below the plasma membrane becomes much higher than the cytosolic Sos1 concentration in the resting cell. This may lead to cross-linking of two Sos1 by a single Grb2 (Figure 9). The additional linking of Sos1 to LAT would increase the stability of Sos1-Grb2-LAT aggregates and thus, the lifetime of Sos1 at the plasma membrane.

## Materials and Methods

### Bioinformatics

Sos family sequences were obtained through a BLAST [45] search against the National Center for Biotechnology Information non-redundant (NCBI-NR) database using the Sos1 sequence from *H. sapien* as a seed and a E-value cutoff of 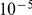. Only completely sequenced proteins were taken, and any sequence that did not belong to the Sos family was removed using phylogenetic analysis. The sequences obtained were aligned with CLUSTAL W [46] and improved manually. Conservation within each group is calculated by identity within each column in the multiple sequence alignment, and three representatives were chosen from each group for Figure 1 using Sequence QR [47]. All the above steps were performed in the Multiseq plugin [48] in VMD [49].

### Binding energy calculations

The structure of the N-SH3 domain and the backbone of the class I peptides were obtained from the NMR structure (PDB ID 1AZE) [21]. The structure of the N-SH3 domain bound to a RP peptide was obtained from a frame at 10 ns of the MD simulation after the RMSD converged. The structure of the C-SH3 domain and the backbone of the RP peptide were obtained from a NMR structure (PDB ID 1IO6) [16]. The structure of the C-SH3 domain and the peptide P1 were obtained from a frame (at 10 ns) in the MD simulation well after the RMSD converged. The structure of the P1 to P4 and RP peptides were based on the backbone of P1 in the above structures and were generated using Scwrl [50].

The protocol for binding the P1 to P4 motifs to C-SH3 required an additional step that utilized MD simulations to generate the conformation for the backbone of the P1 through P4 motifs bound to C-SH3. MD simulations have previously been used to produce good candidate conformations for AutoDock as, for example, in predicting novel inhibitors for RNA-editing ligases [22]. The backbones of the peptide and the receptor molecules were kept rigid during the docking procedure. All polar hydrogen atoms in the receptor and peptide molecules were added using AutoDock. Mass-centered grid maps were generated with 0.375 Å spacing by the AutoGrid program for the whole protein target. AutoDock4 parameters were used for all the atoms during the docking procedure. Lennard-Jones parameters 12–10 and 12–6 were used for modeling H-bonds and van der Waals interactions, respectively. A distance-dependent dielectric permittivity was used for the calculation of the electrostatic grid maps. The Lamarckian genetic algorithm (LGA) was used to predict the binding site and binding energy of the peptide to the SH3 domains. The number of generations was set to 250 million in all runs. Random starting positions on the entire protein surface, random orientations, and side-chain torsions were used for the ligands. The runs were performed with 50000 generations and the population size was set to be 150.

### Molecular dynamics

#### Modeling

The starting structure of the *apo* Grb2 is based on the crystal structure (PDB ID 1GRI) [51]. The missing residues were modeled using MODELLER [52]. In order to ensure that the MD simulation of *apo* Grb2 is not sampling configurational space close to a single energetic minimum [2], a separate simulation of *apo* Grb2 was run at 400 K for 30 ns. The secondary structure Grb2 remains intact throughout this simulation. However, the distance between the two SH3 domains increases with respect to the crystal structure. A frame from this simulation was chosen such that the distance between the two domains is the largest. This structure was cooled down to create a second simulation of *apo* Grb2 at room temperature. The structure was also chosen as the starting point for the Grb2 simulation with peptides P1 and P2. The structure of Grb2 with P1 and P2 on the N-SH3 and C-SH3 domains respectively was modeled using MODELLER [52] with the *apo* Grb2 (high temperature run), P1 bound to N-SH3 domain (PDB ID 1AZE), and a class I peptide bound to C-SH3 domain (PDB ID 1IO6) as templates. The orientation of P2 on C-SH3 was the reverse of the class I peptide on the C-SH3 domain in the NMR structure. In order to model the starting configuration of the N-SH3:RP system, we aligned N-SH3:P1 complex with C-SH3 bound to a class I peptide (PDB ID 1IO6) based on the SH3 domains. We used Modeller to model the N-SH3:RP peptide complex. The same alignment was used to model the C-SH3 domain bound to P1 peptide.

#### Simulation protocol

The MD simulations of the solvated complexes were performed using NAMD2 [53] with the CHARMM27 force field [54]. The proteins were explicitly solvated with TIP3 water molecules [55]. The histidine protonation states were predicted using the PROPKA server and visually checked [56]. Psfgen was used to add hydrogen atoms to the macromolecules. The protein was solvated using the solvate plugin in VMD and potassium ions were placed at the electrostatic minimum to neutralize the system using the Ionize program (http://www.ks.uiuc.edu/Development/MDTools/ionize/) according to the protocol in [57]. The box sizes varied from 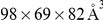 to 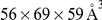 with the number of atoms in the system varying from 51000 to 21250.

All simulations except the high temperature run were done with periodic boundary conditions using the NPT ensemble with pressure set to 1 atmosphere using the Langevin piston and temperature set to 298 K using Langevin dynamics. The high temperature run was performed at 400 K with pressure set to 1 atmosphere. Electrostatics were calculated with the particle mesh Ewald method [58]. The van der Waals interactions were calculated using a switching distance of 10 and a cutoff of 12. Time steps for updates of bonded, van der Waals, and electrostatic calculations were 1, 2, and 4 fs, respectively.

All the systems were minimized using a 4-step protocol in which the water molecules were allowed to associate with the macromolecule before allowing the macromolecule to move. These steps were: heavy atoms fixed (2,000 steps), heavy atoms fixed excluding water and ions (3,000 steps), macromolecule backbone atoms fixed (5,000 steps), and all atoms free to move (20,000 steps). During the initial equilibration, the system was gradually heated to 298 K [57] during which different parts of the system were harmonically constrained. The initial temperature was set to 100 K, and ions and heavy atoms in the protein and nucleic acid chains were harmonically constrained for the first 25,000 fs. Then the temperature was raised to 200 K, and backbone atoms were harmonically constrained for 25,000 fs. Force constants for all harmonic constraints were set to 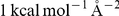. Finally, the temperature was raised to 298 K, and all atoms were freed for the next 0.9 ns. After this 1-ns equilibration, each system was run for a further 399 ns using RATTLE [59] and SETTLE [60] algorithms to constrain hydrogen atoms in the system, and 2 fs timesteps were used in the production run. The coordinates were saved once every ps in these 399 ns.

### Calculation of local concentration 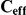



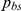
 was calculated from MD simulations of Grb2 bound to the P1 and P2 peptides. The distances between the 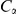 atoms of the appropriate terminii of these two peptides are calculated. The histogram (H(r)) of distance separation (r) is calculated using 100 bins. The probability density is calculated using the formula:
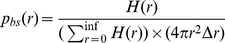
(13)where 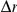 is the width of each interval in the histogram. 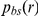 is substituted into Eq. to calculate 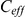.

## Supporting Information

Table S1Effect of different conformations for the SH3 domain and backbone of peptide on binding energies (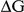 in kcal/mol) estimated using AutoDock.(PDF)

Table S2Effective concentration of motif (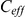 in mM) near the second binding site when another motif is bound to the appropriate SH3 domain of Grb2 using the delta-function approximation. The probability of the distance between the binding sites (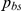) is approximated using a set of delta functions. The delta functions were centered at the distance between the two ends of the motif which depends on the type of motif (class I or class II) and the order they occur in the sequence (see Figure 6). These distances were calculated from the modeled structure of motifs P1 and P2 bound to the N- and C-SH3 domains of Grb2 respectively. The Effective dissociation constant (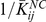 in 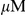) of both motifs in Sos1 binding to Grb2 using the delta function to estimate 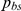 are shown in parenthesis.(PDF)
