# Supplementary material for: Quantifying Intramolecular Binding in Multivalent Interactions: A Structure-Based Synergistic Study on Grb2-Sos1 Complex
Source: PLoS Comput Biol. 2011 Oct 13;7(10):e1002192. doi: 10.1371/journal.pcbi.1002192 (PMC3192808; doi:10.1371/journal.pcbi.1002192)
Supplement: Table S1 — Effect of different conformations for the SH3 domain and backbone of peptide on binding energies ( in kcal/mol) estimated using AutoDock. (PDF) [file pcbi.1002192.s001.pdf]

MD simulation frame	C-SH3-P1	C-SH3-P2	C-SH3-P3	C-SH3-P4	N-SH3-P1
20 ns	-3.2	-3	-1.4	-2.7	-4.9
40 ns	-3.8	-3.7	-2.0	-3.1	-7.3
60 ns	-5.1	-4.4	-3.7	-3.6	-4.6
80 ns	-9.5	-5.7	-5.4	-5.9	-5.2
100 ns	-7.8	-5.7	-5.4	-5.9	-5.0
120 ns	-5.8	-4.5	-3.5	-4.3	-4.3
140 ns	-5.1	-4.9	-4.3	-4.7	-5.1
160 ns	-6.6	-4.5	-4.1	-3.9	-7.0
180 ns	-8.0	-5.4	-4.0	-4.6	-5.8
200 ns	-5.3	-3.5	-2.5	-2.7	-6.2
Mean	-6.0 $\pm$ 2.0	4.6 $\pm$ 1.0	3.6 $\pm$ 1.3	4.2 $\pm$ 1.3	5.5 $\pm$ 1.0

Table S1:
