# Supplementary material for: Quantifying Intramolecular Binding in Multivalent Interactions: A Structure-Based Synergistic Study on Grb2-Sos1 Complex
Source: PLoS Comput Biol. 2011 Oct 13;7(10):e1002192. doi: 10.1371/journal.pcbi.1002192 (PMC3192808; doi:10.1371/journal.pcbi.1002192)
Supplement: Table S2 — Effective concentration of motif ( in mM) near the second binding site when another motif is bound to the appropriate SH3 domain of Grb2 using the delta-function approximation. The probability of the distance between the binding sites () is approximated using a set of delta functions. The delta functions were centered at the distance between the two ends of the motif which depends on the type of motif (class I or class II) and the order they occur in the sequence (see Figure 6). These distances were calculated from the modeled structure of motifs P1 and P2 bound to the N- and C-SH3 domains of Grb2 respectively. The Effective dissociation constant ( in ) of both motifs in Sos1 binding to Grb2 using the delta function to estimate are shown in parenthesis. (PDF) [file pcbi.1002192.s002.pdf]

		Peptide bound to N-SH3 domain				
		P1	P2	P3	RP	P4
Peptide bound to C-SH3 domain	P1	-	1.0 (10.0)	1.2 (14.6)	0.7 (20.9)	0.6 (20.5)
	P2	0.7 (54.4)	-	1.1 (204.2)	0.9 (181.5)	0.7 (163.5)
	P3	1.0 (55.8)	0.8 (87.5)	-	1.2 (167.5)	1.0 (156.5)
	RP	0.7 (95.7)	0.9 (106.9)	1.2 (167.5)	-	0.3 (469.6)
	P4	0.5 (85.7)	0.7 (105.4)	0.9 (154.2)	0.3 (514.0)	-

Table S2: Effective concentration of motif (C_{eff} in mM) near the second binding site when another motif is bound to the appropriate SH3 domain of Grb2. The probability of the distance between the binding sites (p_{bs}) is approximated using a set of delta functions. The delta functions were centered at the distance between the two ends of the motif which depends on the type of motif (class I or class II) and the order they occur in the sequence (see Figure 6). These distances were calculated from the modeled structure of motifs P1 and P2 bound to the N- and C-SH3 domains of Grb2 respectively. Effective dissociation constant (1/ $\bar{K}_{ij}^{NC}$ in μM) of both motifs in Sos1 binding to Grb2 using the delta function to estimate p_{bs} is given in parenthesis.
